# Interventions to improve pharmacists’ competency in chronic disease management: a systematic review of randomized controlled trials

**DOI:** 10.1186/s12909-024-06393-z

**Published:** 2024-12-18

**Authors:** Farida Rendrayani, Auliasari M. Utami, Widya N. Insani, Falerina Puspita, Sofa D. Alfian, Thang Nguyen, Irma M. Puspitasari

**Affiliations:** 1 Department of Pharmacology and Clinical Pharmacy, Faculty of Pharmacy, Universitas Padjadjaran, Sumedang, Indonesia; 2 Center of Excellence for Pharmaceutical Care Innovation, Universitas Padjadjaran, Sumedang, Indonesia; 3 Center for Health Technology Assessment, Universitas Padjadjaran, Sumedang, Indonesia; 4 Can Tho University of Medicine and Pharmacy, 179 Nguyen Van Cu, An Khanh Ward, An Khánh, Cần Thơ City, Vietnam

**Keywords:** Intervention, Pharmacists, Competency, Chronic disease, Disease management

## Abstract

**Introduction:**

Effective chronic disease management (CDM) is vital for addressing chronic disease challenges. Given the importance of ensuring pharmacists’ competence in CDM, interventions targeting knowledge, skills, and attitudes are essential. Therefore, a comprehensive and up-to-date study is needed to analyze these interventions’ effect and potential development. Categorizing the interventions based on the Effective Practice and Organization of Care (EPOC) taxonomy is essential for better informing policymakers. The objectives of this systematic review were to identify interventions to improve pharmacists’ competency in chronic disease management based on the EPOC taxonomy and summarize their effectiveness.

**Methods:**

Following methods in the Cochrane Handbook, a systematic search was conducted up to April 2024 on MEDLINE and Scopus. The inclusion criteria were an intervention study with a randomized controlled trial (RCT) design published in English, targeting pharmacists, and measuring knowledge, skills, and attitudes in aspects of CDM. The risk of bias was assessed using Cochrane’s RoB 2 tool for either randomized or cluster-randomized trials. Findings are reported narratively and align with the Preferred Reporting Items for Systematic Reviews and Meta-Analyses (PRISMA) statement.

**Results:**

We included 11 RCT studies that focused on various aspects of CDM among community and hospital pharmacists. Implementation strategies and combined implementation strategies–delivery arrangements interventions were identified. Six implementation strategies interventions consistently yielded effective results, with scores ranging from 0.99 to 9.17 (*p* < 0.05). However, the other two implementation strategies interventions reported mixed results, with no significant improvements in knowledge or skills. Two implementation strategies-delivery arrangements interventions showed improvements, with score differences ranging from 4.5% (95% CI: 1.6%-7.4%) to 30% (95% CI: 29%-40%). Conversely, one implementation strategies-delivery arrangements intervention showed no significant improvement. The risk of bias assessment revealed varying levels of bias across the studies.

**Conclusions:**

Implementation strategies and combined implementation strategies–delivery arrangements interventions improved pharmacists' competency in CDM. Most interventions consistently resulted in significant improvements in pharmacists' knowledge, skills, and attitudes. These findings underscore the potential of tailored, competency-based interventions to improve pharmacist competencies in CDM. Policymakers can use these insights to create guidelines and policies that promote ongoing professional development for pharmacists.

**Supplementary Information:**

The online version contains supplementary material available at 10.1186/s12909-024-06393-z.

## Introduction

Chronic diseases contribute significantly to death rates, disability, and reduced quality of life, posing a challenge to health systems around the globe [1–3]. The long duration and slow progression of this disease lead to increased burdens on individuals, societies, and economies [1, 4, 5]; thus, requiring comprehensive and specialized management approaches [1, 4]. Studies suggest that effective chronic disease management (CDM), including health promotion, detection, screening, surveillance, self-management support, treatment, care, rehabilitation, and palliative care, could address this issue [6–8].

Pharmacists are pivotal in CDM since they frequently serve as the first contact for patients seeking medication and health advice [9, 10]. They can provide patients with appropriate medications, assist with medication adherence, and provide guidance on lifestyle modifications [11, 12]. The pharmacist, however, must maintain essential competencies, including knowledge, skills, and attitudes [13], to achieve effective chronic disease management.

In professional practice, combining knowledge, skills, and attitudes is important. Having a good understanding helps in making good decisions [14]. A positive attitude facilitates collaboration with other workers and promotes continuous improvement [15]. Furthermore, skills allow the use of knowledge and attitudes in real-world situations, ensuring that tasks are accurately and efficiently performed [16, 17]. According to the theory of planned behavior, these factors together influence professionals' intentions and actions, leading to improved practices and outcomes [18]. Developing these competencies is critically important for achieving high-quality and sustained results.

Various studies have examined interventions to improve pharmacists’ competency in chronic disease management [19–21]. Nonetheless, a comprehensive and up-to-date study is needed to analyze these interventions’ effects and their potential development. This study may be useful to decision-makers in designing appropriate and effective interventions for improving pharmacists' competence in managing chronic diseases. Categorizing interventions using the Effective Practice and Organization of Care (EPOC) taxonomy provides better decision-making support because it ensures consistent classification and comparability [22–24]. To the best of our knowledge, no such comprehensive study is available at this time. Therefore, the objectives of this systematic review were to identify interventions to improve pharmacists’ competency in chronic disease management based on the EPOC taxonomy and summarize their effectiveness.

## Methods

We conducted a systematic review following the Cochrane Handbook for Systematic Reviews of Interventions [25] and the York Centre for Reviews and Dissemination [26] guidelines. We reported our review by following the Preferred Reporting Items for Systematic Reviews and Meta-Analyses (PRISMA) statement [27]. Tables S1 and S2 in the supplementary materials contain details of the PRISMA checklist. We did not register the review protocol because our outcome was pharmacists’ competency, since the registration platform only accepts reviews with outcomes directly related to human health [28].

## Study selection criteria

### Types of studies

We searched for original research studies published in English using a randomized controlled trial (RCT) design. An RCT is a scientific experiment where participants are randomly assigned to the intervention or the control group, minimizing selection bias and ensuring comparability; thus, providing high-quality evidence of treatment effectiveness [25, 29]. We excluded gray literature (e.g., theses and dissertations), letters to the editor, commentaries, conference proceedings, case reports, study protocols, review articles, and non-English publications.

### Types of participants

Studies that involve pharmacists, either in community or hospital settings, were included. While the participants did not need to be solely pharmacists, the responses from the pharmacists should be separate from those of the other participants.

### Types of intervention

Our study included a comprehensive range of interventions to enhance pharmacists’ competency in chronic disease management. Competency was defined as the combination of knowledge, skills, and attitudes [30, 31], whereas chronic diseases were defined as persistent conditions with lasting effects, requiring ongoing medical attention or limiting daily activities for a year or more [32]. These included conditions such as heart disease, cancer, diabetes, arthritis, asthma, stroke, Alzheimer’s disease, obesity, and addiction [32–36], which were diagnosed either by a physician or self-reported.

With respect to CDM aspects, our inclusion criteria covered studies related to health promotion, detection, screening, surveillance, self-management support, treatment, care, rehabilitation, and palliative care [6]. We did not restrict the intervention characteristics, such as delivery mode, format, frequency, or duration.

Using the latest EPOC taxonomy [37], the interventions were further categorized into four types:

1. Delivery Arrangements: This domain covers interventions that focus on how healthcare services are organized and delivered, which could involve team-based care, case management, providers’ coordination and communication, and continuity of care.
2. Financial Arrangements: This domain includes interventions that focus on how economic factors affect healthcare, such as funding, payment models, and financial incentives.
3. Governance Arrangements: The interventions involve policy, leadership, and accountability structures within healthcare systems, including accreditation and certification, regulations and guidelines, and accountability mechanisms.
4. Implementation Strategies: Interventions in this domain focus on strategies for implementing healthcare interventions, for example, training for healthcare providers, audits, and feedback mechanisms.

Additional details on these types can be found on the EPOC website [37].

### Types of comparators

We did not restrict the type of comparator or control group in this study. The control groups could receive no intervention, a different intervention, or usual care.

### Types of outcomes

We included studies that measured competency; therefore, it should measure at least one of these three aspects: knowledge, skills, or attitudes [30, 31]. Generally, knowledge is the accumulation of information, insights, and experiences that enable individuals or groups to comprehend a specific issue [38]. Skill is characterized as proficiency in performing a task or problem-solving within a certain amount of time or energy [39]. On the other hand, attitude is described as the tendency to evaluate things in a particular manner, whether positive, negative, or neutral, which influences behavior and decision-making [40].

We focused only on these foundational competencies because they are essential for effective chronic disease management. While practice is important, it is often influenced by external factors such as the workplace environment, resources, and organizational policies, which can vary widely. Our results are derived solely from interventions that do not include practice, allowing for direct comparisons and adaptable insights across various contexts. We also excluded studies that solely measured the following:

1. intervention feasibility or acceptance;
2. organizational outcomes, such as the success rate of treatment, the number of cases detected, or the level of patient satisfaction with services;
3. patient outcomes, including clinical, humanistic, and economic aspects.

## Search methods for identifying studies

A systematic search was conducted up to April 2024, utilizing pre-established strategies to identify relevant studies in the MEDLINE and Scopus databases. These databases are widely acknowledged as primary sources for identifying relevant references and reducing bias in systematic reviews of health interventions [41, 42]. We identified original studies that met the inclusion criteria during this search. We used Boolean operators such as “OR” to expand the exploration within each concept and “AND” to refine the findings. Additionally, Medical Subject Headings (MeSH) terms were applied in the MEDLINE search, with the detailed search terms outlined in Table 1.

**Table 1:** Search strategy in database searching

Database	Search terms
MEDLINE	("pharmacists"[MeSH Terms] OR "pharmacist*"[Title/Abstract] OR "pharmacies"[MeSH Terms] OR "pharmacy*"[Title/Abstract]) AND ("randomized controlled trials as topic"[MeSH Terms] OR "randomized controlled trial*"[Title/Abstract] OR "randomised controlled trial*"[Title/Abstract]) AND ("competency”[Title/Abstract] OR “competency-based education”[MeSH Terms] OR “knowledge"[Title/Abstract] OR "attitude"[Title/Abstract] OR "attitude of health personnel"[MeSH Terms] OR "skill*"[Title/Abstract])
Scopus	(pharmacist* OR pharmacy*) AND (“randomized controlled trial*” OR “randomised controlled trial”) AND (competency OR knowledge OR attitude* OR skill*)

In addition to the database searches, supplementary hand searches were conducted in healthcare journals and Google Scholar [25, 41]. Citation chasing was also employed by tracing articles referencing the identified studies (forward) and thoroughly reviewing the reference lists (backward) [25].

## Data collection and analysis

### Study selection

The search results were exported to Zotero version 6.0.13 (Corporation for Digital Scholarship, Vienna, USA), and any duplicates were thoroughly identified. Two researchers (FR and AMU) independently conducted the screening process, starting with a thorough review of titles and abstracts. The full texts of the selected articles were examined for accuracy, and discrepancies were resolved through discussion.

### Data extraction

The data from the included studies were manually extracted into a predefined format in Microsoft® Excel® 2019 MSO version 2210 (Microsoft Corporation, Redmond, WA, USA). Two reviewers (FR and AMU) independently extracted the data. Any disagreements were addressed through collaborative discussions with other researchers. Attempts were made to email the corresponding authors if any data were incomplete. The extracted data from each study included author, publication year, country of the study, funding, study design, participants, sample size, chronic disease type, CDM aspect, intervention type, intervention description, comparator/control, outcome, outcome measurement method, follow-up duration, and effectiveness.

### Risk of bias assessment

Using two distinct tools—the Revised Cochrane Risk of Bias tool for randomized trials (RoB 2) and the Risk of Bias tool for cluster-randomized trials (RoB 2 CRT) [25]—three reviewers (FR, WNI, and FP) independently evaluated the studies’ risk of bias. A study was classified as having a low risk of bias if all domains demonstrated low risk. If ‘some concerns’ were noted in at least one domain without reaching a high-risk threshold, the study was categorized as having some concerns. Furthermore, studies possessing ‘high bias risk’ showed high risk in at least one domain or ‘some concerns’ across multiple domains, which substantially undermined confidence in the results [25]. We resolved any discrepancies through discussion.

### Data synthesis

Descriptive statistics were employed to outline the characteristics of the included studies. Owing to differences in the study's methodology, we synthesized the data narratively. During the synthesis process, researchers were involved in discussions about the findings. The synthesis aligns with the narrative synthesis guidelines outlined by the York Centre for Reviews and Dissemination for systematic reviews [26].

## Results

### Literature search results

**Fig. 1:**
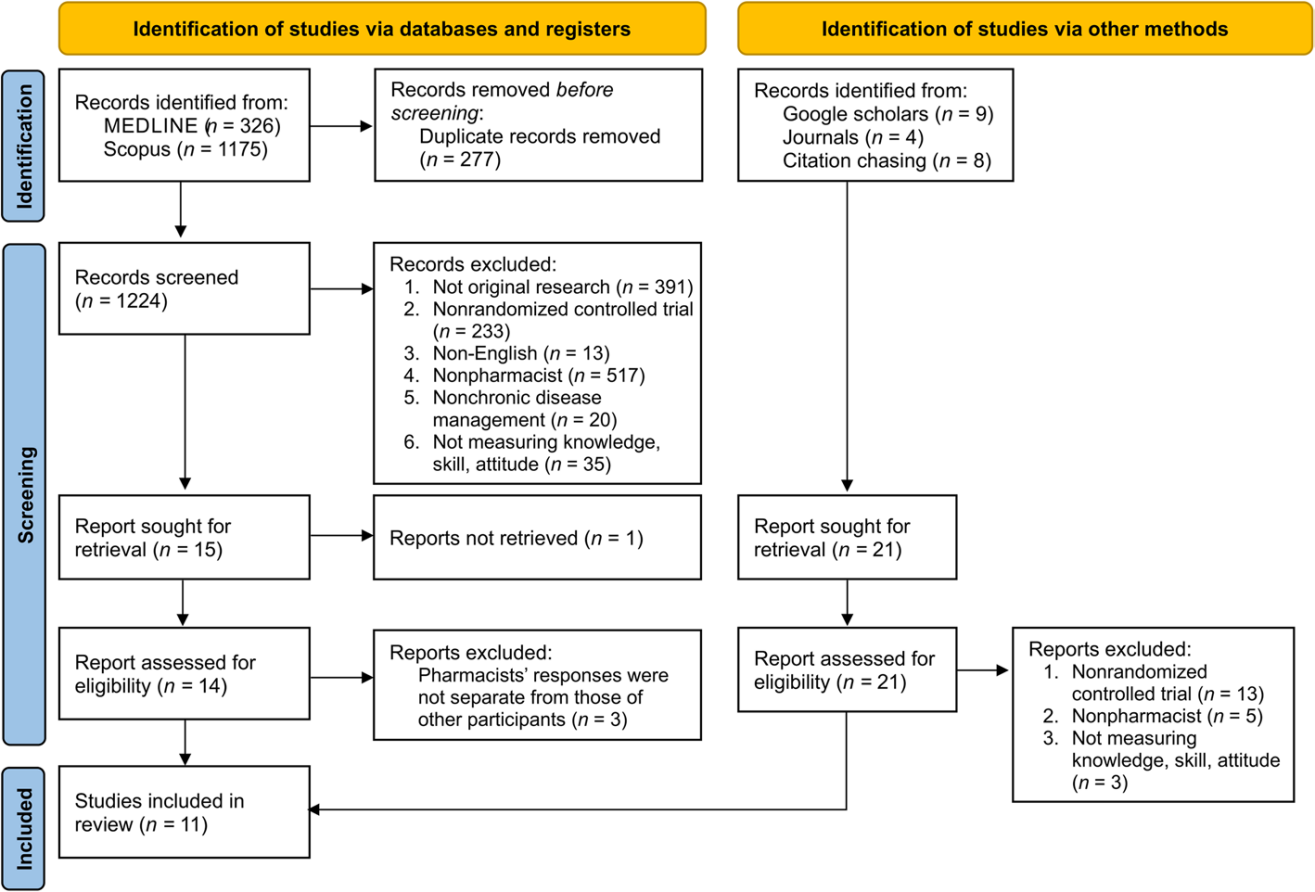
The study selection process

We found 326 records in MEDLINE and 1,175 in Scopus. After removing 277 duplicates, we screened the titles and abstracts of 1,224 records and selected 15 articles for full-text evaluation. One article [43] could not be accessed, and we could not contact the authors to obtain the full text. Additionally, we excluded three other articles [44–46] since the pharmacists’ responses were not separate from those of the other participants. No additional suitable articles were found despite manual searches in journals, Google Scholar, or citation chasing. Finally, 11 articles [19–21, 47–54] met the inclusion criteria and were included in the final review. Figure 1 depicts the flowchart of the study selection process.

## Characteristics of the included studies

Table 2 summarizes the characteristics of the included studies. These studies were published between 1999 and 2024, and only six studies (54.5%) were published within the last decade [19–21, 47–49]. Nine studies were conducted in high-income countries, including Qatar, the United States, Japan, and Canada, whereas two were conducted in upper-middle-income countries, including China and Iran.

**Table 2:** Characteristics of the included studies

Author, Year	Country	Funding	Study Design	Participants	Sample Size	Chronic Diseases	CDM Aspect
Shen et al., 2024 [19]	China	Wuhan University, Chinese Pharmaceutical Association Hospital Pharmacy Department, and Huazhong University of Science and Technology	RCT	Community pharmacists	• Allocated: IG = 12; CG = 12 • Completed: IG = 12; CG = 12	Cardiovascular and cerebrovascular diseases, cancer, and chronic respiratory diseases	Treatment
El Hajj et al., 2022 [20]	Qatar	Qatar University Office of Research and Graduate Studies	RCT	Community pharmacists	• Allocated: IG = 77; CG = 87 • Completed: IG = 54; CG = 32	Tobacco addiction	Health promotion
Haga et al., 2021 [47]	United States	US National Institutes of Health	Cluster-RCT	Community pharmacists	• Allocated: IG = 21; CG = 15 • Completed: IG = 9; CG = 7	Mental health disorders, pain, and cardiovascular diseases	Detection
Fujii et al., 2021 [48]	Japan	JSPS KAKENHI	RCT	Community pharmacists	• Allocated: IG = 60; CG = 60 • Completed: IG = 59; CG = 56	Schizophrenia	Care
Lalonde et al., 2017 [21]	Canada	Canadian Institutes of Health Research, Pfizer Canada Inc., LEO Pharma, and Amgen Inc	Cluster-RCT	Community pharmacists	• Allocated: IG = 345; CG = 149 • Completed: IG = 200; CG = 123	Chronic kidney disease	Treatment
Liekens et al., 2014 [49]	Belgium	None	Cluster-RCT	Community pharmacists	• Allocated: IG = 21; CG = 19 • Completed: IG = 21; CG = 19	Depression	Care
Sarayani et al., 2012 [50]	Iran	None	RCT	Community pharmacists	• Allocated: IG-1 = 60; IG-2 = 60; CG = 60 • Completed: IG-1 = 35; IG-2 = 40; CG = 42	Obesity	Self-management support
Legris et al., 2011 [51]	Canada	Cercle du Doyen of the Faculty of Pharmacy of the Universite de ´ Montreal and Amgen Canada Inc	RCT	Community pharmacists	• Allocated: IG = 52; CG = 18 • Completed: IG = 49; CG = 18	Chronic kidney disease	Treatment
Lalonde et al., 2008 [52]	Canada	Fonds de la recherche en sante´ du Que´bec, Bourse du Cercle du Doyen,Pfizer Canada Inc.,Amgen Canada Inc., Bristol-Myers Squibb/Sanofi-Synthelabo, Hoffmann-La Roche Limite´e, LEO Pharma Inc., Merck Frosst Canada & Co, Pharmaceutical Partners of Canada Inc., Pro Doc Lte´e, Sabex, and Shire BioChem Inc	Cluster-RCT	Community pharmacists	• Allocated: IG = 50; CG = 51 • Completed: IG = 36; CG = 45	Chronic kidney disease	Treatment
Dolovich et al., 2007 [53]	Canada	Merck Frosst Canada Inc	RCT	Community pharmacists	• Allocated: IG = 33; CG = 31 • Completed: IG = 29; CG = 30	Asthma	Care
Jackevicius & Chapman, 1999 [54]	Canada	Canadian Society of Hospital Pharmacists Research and Education Foundation	RCT	Hospital pharmacists	• Allocated: IG = 25; CG = 25 • Completed: IG = 23; CG = 19	Asthma and chronic obstructive pulmonary disease	Self-management support

*RCT* randomized control trial, *IG* intervention group, *CG* control group, *IG-1* intervention group 1, *IG-2* intervention group 2 (three-arm RCT)

The 11 included studies employed various study designs: seven were RCTs—six with two arms [19, 20, 48, 51, 53, 54] and one with three arms RCT [50]—and four were two-arm cluster-RCTs [21, 47, 49, 52]. Most studies focused on community pharmacists, except one by Jackevicius and Chapman (1999), who targeted hospital pharmacists. Among the included studies, only four [19, 48, 50, 54] had equal sample sizes between the intervention and control groups. The sample sizes of these studies ranged from 12 [19] to 60 [50] participants per group. In studies with uneven group sizes, the number of pharmacists in the intervention groups varied from 21 [47, 49] to 345 [21].

The studies analyzed interventions in various aspects of CDM. Four studies [19, 21, 51, 52] focused on the treatment of cardiovascular and cerebrovascular diseases, cancer, chronic respiratory diseases, and chronic kidney disease. El Hajj et al. (2022) examined intervention in health promotion for tobacco addiction, whereas Haga et al. (2021) focused on the detection of mental health disorders, pain, and cardiovascular diseases. Three studies [48, 49, 53] focused on the care aspect of schizophrenia, depression, and asthma. Additionally, two studies [50, 54] focused on self-management support for obesity and asthma.

## Intervention types and their effectiveness

Table 3 presents a summary of the interventions and their effectiveness. The types of interventions in the included studies were implementation strategies (*n* = 8) [19, 20, 48–51, 53] and a combination of implementation strategies and delivery arrangements (*n* = 3) [21, 47, 52]. The implementation strategies interventions involved diverse educational strategies, including educational materials and interactive educational meetings with problem-based exercises, role play, discussion, observation, and simulation. On the other hand, the delivery arrangements interventions involved coordination of care amongst different providers, communication between providers, continuity of care, and the use of information and communication technology. Six studies (54.5%) had control groups receiving less effective interventions, four had usual care controls, and one had no intervention control [53].

**Table 3:** Summary of the interventions and their effectiveness

Author, Year	Intervention		Comparator /Control	Outcome	Measurement Method	Follow-up Duration	Effectiveness
	**Type**^**a**^	**Description**					
Shen et al., 2024 [19]	Implementation Strategies	A 12-h training course covering prescription checking, rational use of medicines, and first aid skills, using the Bridge-In, Objective, Preassessment, Participatory Learning, Post-assessment, and Summary (BOPPPS) model	Didactic lecture on a similar topic	Knowledge and skills	Single and multiple-choice questions, prescription analysis, and practical skills assessment; postintervention	-	Significant knowledge and skills improvement in the intervention group, with total score differences of 9.17 (*p* < 0.001) and 6.5 (*p* < 0.01), respectively
El Hajj et al., 2022 [20]	Implementation Strategies	Four-day tobacco cessation education including didactic lectures, problem-based exercises, role plays, and simulations	Didactic lecture on women’s health and contraception	Skills	Six-station Objective Structured Clinical Examination (OSCE) with standardized patients, scored by a trained, blinded assessor; postintervention	-	Significant skills improvement in the intervention group across all cases, with adjusted mean score differences of 7.2, 9.6, 7.1, 4.9, 8.2, and 4.5 (*p* < 0.001) for cases 1–6, respectively
Fujii et al., 2021 [48]	Implementation Strategies	One-hour lecture on schizophrenia, group discussions, and interviews with patients	Didactic lecture on schizophrenia only	Attitudes	Eight statements on a 5-point scale; pre and postintervention	-	Significant attitude improvement in the intervention group with an effect size of 0.49 (*p* < 0.001)
Liekens et al., 2014 [49]	Implementation Strategies	A one-day course including lectures on depression, treatment, and basic communication; discussions with consumer educators; and practice through role-playing with simulated patients and observing colleagues	Usual care	Skills and attitudes	Seven statements on a 5-point scale, rated by trained mystery shoppers; follow-up	8 months	Significant skills and attitude improvement in the intervention group, with mean score differences of 3.29 (*p* = 0.049) and 0.99 (*p* = 0.031), respectively
Sarayani et al., 2012 [50]	Implementation Strategies	The lecture plus case-discussion arm consisted of didactic mini-lectures with interactive case discussions (240 min); the lecture plus small-group training arm consisted of didactic mini-lectures followed by interactive workshops with clinical pharmacy residents (265 min)	Didactic lecture only (240 min) on a similar topic	Knowledge and attitudes	Eighteen multiple-choice questions, two case vignette essays, and eight statements on a 5-point scale; preintervention, postintervention, and follow-up	4 weeks	1. No significant knowledge and attitude improvement in any group from pre to postintervention 2. Significant knowledge improvement in the lecture plus small group training from preintervention to follow-up, with an effect size of 0.58 and mean scores differences of 11.30 (*p* = 0.002) with lecture only and 13.40 (*p* = 0.001) with lecture plus case discussion 3. No significant attitude improvement in any group from preintervention to follow-up
Legris et al., 2011 [51]	Implementation Strategies	A 60-min interactive web-training with clinical vignettes on identifying and managing drug-related problems in CKD	Usual care	Knowledge and skills	Ten multiple-choice questions and two clinical vignettes; pre and postintervention	-	Knowledge and skills improvement in the intervention group, with adjusted incremental changes of 22% (95% CI: 16%-27%) and 24% (95% CI: 16%-33%), respectively
Dolovich et al., 2007 [53]	Implementation Strategies	A one-day workshop with didactic teaching, group work, and role-playing − focusing on asthma and its therapies, expiratory monitoring and inhaler use, and phone follow-up of patients	No intervention	Skills	Three cases were assessed through simulated patient visits using the Global Rating Scale; follow-up	3–5 weeks	Significant skills improvement in the intervention group in two of the three cases presented with mean score differences of 1.17 (*p* = 0.043) in case 1 and 1.16 (*p* = 0.01) in case 3
Jackevicius & Chapman, 1999 [54]	Implementation Strategies	One-hour workshop including interactive activities and practice of inhaler techniques	A 15-min review of manufacturer’s package inserts	Knowledge and skills	Six true/false and five short answer questions, and inhaler technique skills demonstration, were assessed by a blinded research assistant; preintervention, postintervention, and follow-up	3 months	1. Significant knowledge and skills improvement in the intervention group from pre to postintervention, with a mean overall score difference of 1.38 (*p* < 0.001) 2. No significant improvement from preintervention to follow-up
Haga et al., 2021 [47]	Implementation Strategies and Delivery Arrangements	In-person training on pharmacogenetics (PGx), additional training on medication therapy management (MTM), and integration of PGx testing with MTM within three months	In-person training sessions only and provision of PGx testing without MTM	Knowledge	Seven multiple-choice questions; pre and postintervention	-	No significant knowledge improvement in any group
Lalonde et al., 2017 [21]	Implementation Strategies and Delivery Arrangements	A 90-minute interactive web-training with clinical vignettes, a clinical guide, and a consultation via phone or forum with chronic kidney disease (CKD) clinic pharmacists within a 12-month study period	Usual care	Knowledge and skills	Ten multiple-choice questions and two clinical vignettes; pre (T0) and postintervention (T12)	-	Knowledge and skills improvement in the intervention group, with adjusted incremental changes of 4.5% (95% CI: 1.6%-7.4%) and 7.4% (95% CI: 3.5%-11.3%), respectively
Lalonde et al., 2008 [52]	Implementation Strategies and Delivery Arrangements	Three-hour workshop including lectures, group discussions on CKD drug therapy, interaction with predialysis professionals, and a communication-network program within a six-month study	Usual care	Knowledge	Four multiple-choice questions on pharmacotherapy, five on dosage adjustments in CKD, and a case study; preintervention and postworkshop	-	Knowledge improvement in the intervention group, with a mean score change of 34% (95% CI: 29–40%)

^a^Based on the Cochrane Effective Practice and Organization of Care (EPOC) taxonomy[37]

Among the studies, seven measured knowledge [19, 21, 47, 50–52, 54], seven [19–21, 49, 51, 53, 54] measured skills, and only three [48–50] measured attitudes. The studies measured knowledge via multiple choice, short answer, or true/false questions; skills via practical assessment/demonstrations, clinical vignettes, Objective Structured Clinical Examination (OSCE), simulated patients, or mystery shoppers; and attitudes via statements on a 5-point scale. Five studies measured the outcomes at preintervention and postintervention, two at immediate postintervention, two at follow-up only (one at 3–5 weeks and the other at 8 months), and two at preintervention, postintervention, and follow-up (one at 4 weeks and the other at 3 months). The studies mostly reported effectiveness based on point or percentage scores, along with *p-*values or 95% confidence intervals (CIs); only two reported effect sizes [48, 50].

Among the studies with an implementation strategies intervention type, six [19, 20, 48, 49, 51, 53] consistently demonstrated effective results in improving pharmacists’ knowledge, skills, or attitudes. Specifically, improvements were associated with differences in point scores ranging from 0.99 (*p* = 0.031) [49] to 9.17 (*p* = 0.001) [19] and percentage scores of 22% (95% CI: 16%-27%) and 24% (95% CI: 16%-33%) [51], as well as effect sizes of 0.49 [48]. However, two studies [50, 54] reported mixed effectiveness results. Significant improvements were reported for knowledge and skills immediately after the intervention, with a point score difference of 1.38 (*p* < 0.001) [54], and for knowledge at the four-week follow-up, with an effect size of 0.58 [50]. No improvements were reported in knowledge or skills at the three-month follow-up [54]. Additionally, there were no improvements in knowledge immediately after the intervention or in attitudes at any observation time [50].

For studies with combined implementation strategies–delivery arrangements intervention type, two showed improvements in knowledge and skills. The studies reported improvements, with percentage score differences ranging from 4.5% (95% CI: 1.6%-7.4%) [21] to 30% (95% CI: 29%-40%) [52]. In contrast, one study demonstrated no significant change in knowledge scores for either the intervention or the control groups.

## Risk of bias

Among seven RCTs, three studies [19, 20, 51] exhibited a low overall risk of bias, while four [48, 50, 53, 54] showed some concerns. The most frequent bias was in the selection of reported results (D5), observed in 50% of RCTs. Bias due to missing outcome data (D3) and outcome measurement (D4) consistently posed low risk across all RCTs. In the four cluster-RCTs, one study [47] had a low overall risk of bias, two [21, 49] showed some concerns, and one [52] exhibited a high risk of bias. The bias was mainly in the reported results selection (D5), as observed in 75% cluster-RCTs. In addition, bias from the timing of participant identification and recruitment in relation to randomization (D1b) and deviations from intended intervention (D2) demonstrated consistently low risk. Further details on the bias assessment are available in the supplementary materials (Tables S3 and S4).

## Discussion

This systematic review identifies implementation strategies and combined implementation strategies–delivery arrangements interventions as interventions implemented to improve pharmacists' competency in CDM. Most of the included studies reported that these interventions effectively improved pharmacists' knowledge, skills, and attitudes, as shown by significant changes in point scores, percentage increases, or notable effect sizes. However, one study did not report effectiveness, whereas two studies reported different results during the follow-up.

## Implementation strategies interventions

Implementation strategies interventions are the most common type of intervention used to improve pharmacists' competency in chronic disease management. These interventions significantly affected the pharmacists’ knowledge, skills, and attitudes. This is likely due to the use of experiential and interactive learning methods [19, 20, 48–51, 53, 54], as well as patient-centered approaches [48], in addition to traditional lectures.

Shen et al. (2024) and Legris et al. (2011) discovered that interactive learning helps with effective knowledge improvement. This is because this method allows more engagement [55]. Other interactive methods, including role-playing, problem-based exercises, and simulation, are also involved in improving pharmacists' skills [19, 20, 49, 51, 53]. These approaches provide pharmacists with hands-on experience and real-life scenarios, enhancing their practical abilities and confidence [56]. The improvements in attitudes of Fujii et al. (2021) and Liekens et al. (2014) could also be attributed to interactive discussions, role-playing, and patient interview approaches. Similarly, a previous study suggested that patient-centered approaches, including contact-based interventions, effectively improved attitudes [57]. Sarayani et al. (2012) also confirmed that interventions lacking a patient-centered approach did not improve pharmacists' attitudes.

Findings from studies measuring short-term (immediately after intervention) and long-term (at the follow-up) effects suggest different results. The short-term effectiveness without long-term retention observed in Jackevicius and Chapman’s study may result from initial engagement and memorization without deep learning [58]. Conversely, the long-term effectiveness without immediate results in Sarayani et al.'s study might be due to the slow process of learning and practicing new skills [58]. These differences could also be due to varied cognitive mechanisms in knowledge acquisition and retention [58], as well as differences in intervention duration, methods (practical demonstrations versus workshops), and follow-up periods.

These findings suggest that both the short-term and long-term effects of implementation strategies interventions should be considered. According to Kim et al. (2013), regular skill practice and the use of external aids or tutors can improve the interventions' effectiveness, including skill retention. These methods help transition declarative skills to the procedural stage, reduce decay, and improve retention time [58]. Therefore, implementation contexts (workplace support systems and the integration of new practices into daily routines), reinforcement, and ongoing skill-building support are key to sustaining effectiveness [59].

## Combined implementation strategies–delivery arrangements interventions

Combined implementation strategies–delivery arrangements interventions were used in three studies. In addition to employing methods similar to previous implementation strategies interventions, these interventions incorporate coordination and communication between providers and the use of information and communication technology. By involving various healthcare providers, the interventions provided a more comprehensive and integrated approach to managing chronic diseases. However, the results of these combined interventions are inconsistent.

Lalonde et al. (2017) reported notable improvements in knowledge and clinical skills, which were probably due to continuous learning and skill development over 12 months—the longest study period observed. Additionally, the interprofessional collaboration employed offered various viewpoints, supporting a well-rounded approach to patient care [60, 61]. Despite a six-month study period, Lalonde et al. (2008) measured outcomes immediately after the workshop session and demonstrated a knowledge improvement of more than 30%. Nonetheless, Haga et al. (2021) reported different results with no significant change in knowledge after three months.

The variability in effectiveness might be caused by differences in intervention delivery methods and implementation contexts (e.g., healthcare settings, levels of multidisciplinary integration, and specific chronic diseases managed) [62, 63]. Other factors may involve challenges in implementing organizational change, resistance to new practices, or inadequate intervention duration. Thus, the level of support and resources available to facilitate multidisciplinary collaboration is an important factor in this intervention type [63].

## Comparisons to other studies

Our findings align with those of previous systematic reviews [64–66], showing that implementation strategies and delivery arrangements interventions are the most widely applied intervention types for improving healthcare professionals’ competency in chronic disease management. A systematic review highlighted that implementation strategies interventions effectively enhance health professionals’ communication skills in promoting health behavior, particularly six to 12 months after the intervention, compared with immediately after or over a longer period [64]. The study, like ours, indicated that combining educational and practical training elements is more effective. Joshi et al. (2014) also reported that combining implementation strategies and delivery arrangements interventions improved health providers’ knowledge and practical skills in managing hypertension and diabetes. Kok et al. (2015) found that while implementation strategies improved providers’ knowledge and skills, there was no reported impact of delivery arrangements on these outcomes. Additionally, the study noted that financial arrangements, such as incentives, are also used, impacting providers’ motivation in managing HIV/AIDS and tuberculosis medication [66].

Our study found that interventions generally focus on implementation strategies and delivery arrangements to improve pharmacist competence in CDM. Both types of interventions are effective in directly improving pharmacists' knowledge, skills, and attitudes in daily practice. The absence of financial and governance arrangements types in the included studies is likely due to their primary focus at the system level, where they aim to enhance organizational compliance and policy implementation rather than directly developing individual skills [67–69]. In line with our findings, Joshi et al. (2014), and Kok et al. (2015) show that delivery and financial arrangements are often combined with implementation strategies for better outcomes. This suggests that combining various interventions, including financial and governance arrangements, can more effectively support the improvement of pharmacist competencies.

## Study strengths, limitations, and implications

This study provides the first comprehensive review of interventions to improve pharmacists’ competency in chronic disease management. Despite variations in intervention types, study populations, and outcome measures, they collectively offer valuable insights into effective strategies. This study highlights the effectiveness of implementation strategies interventions and the potential benefits of combining implementation strategies and delivery arrangements approaches in the continuing education and professional development of pharmacists. The detailed breakdown of intervention types and their specific outcomes offers a nuanced understanding of their impact on pharmacist competency.

This review’s strength also lies in not restricting publication years, which allows us to gather as much relevant study as possible to track development in intervention approaches over time. Across the 25-year span of the studies reviewed, we found a clear shift in educational approaches from content-based to competency-based learning. This emphasizes hands-on experiences that enhance pharmacists’ understanding and retention in real-world contexts. Implementation science has also become essential in evaluating teaching methods. With an evidence-based approach, recent studies use standardized tools, such as the OSCE—used in the El Hajj et al. (2022) study—to more accurately measure clinical skills and support evidence-based curriculum recommendations. On the other hand, technology—such as online learning and educational applications [21, 47]—has expanded access and supports personalized learning, allowing students to learn anytime and anywhere. This review also identifies a notable trend toward practical skills development, with more recent studies using simulation, OSCE, and role-play to prepare pharmacists for the challenges of the workforce [19, 20, 48, 49], which was less visible in earlier studies. Although the data do not indicate a direct increase in effectiveness over time, newer practical, technology-based approaches align closely with today's educational needs. The key takeaway from these findings is the importance of adapting learning methods to technological advances and evolving healthcare needs. Modern topics, such as tobacco cessation and pharmacogenetics [20, 47], are now more widely taught, reflecting the need to align educational materials with public health demands.

However, it is important to consider several limitations when interpreting the findings. First, the heterogeneity of interventions and outcome measures, along with predominantly high-income country settings, may restrict the generalizability of findings across different contexts and populations. Certainty of evidence also varied across studies, with some exhibiting a higher risk of bias, particularly concerning bias in selecting reported results. Nevertheless, the overall quality of evidence was moderate. Moreover, the lack of long-term follow-up assessments hinders conclusions regarding the sustainability of intervention effects. Furthermore, publication bias may exist because we did not search gray literature—only peer-reviewed published articles were included to ensure comparability of study quality. Our study also excluded some interventions that might be designed to indirectly improve knowledge, skills, and attitudes, through enhancing delivery arrangement processes or patient outcomes, which could result in the absence of complex, multifaceted interventions with significant indirect benefits.

To address the limitations, future research should focus on developing interventions tailored to low- and middle-income countries facing a growing burden of chronic diseases. Studies must also enhance methodological quality to strengthen evidence certainty and assess long-term follow-up impacts to evaluate intervention sustainability. Exploring innovative approaches such as technology-based interventions and interprofessional collaboration is essential for enhancing effectiveness and scalability. Additionally, research should explore financial and governance arrangements interventions types for chronic disease management while evaluating interventions' cost-effectiveness to enhance pharmacists’ competency in this area.

This review has several implications for pharmacy practice. Healthcare administrators and educators can use this information to design more effective, competency-based training programs aligned with current needs. The programs should include the most effective elements of implementation strategies and delivery arrangements interventions, such as combining didactic teaching with interactive, patient-centered approaches, and communication programs. Encouraging collaboration between professionals and the use of technology-based interventions may make such interventions more successful and scalable. Policymakers can use these findings to develop guidelines and policies that facilitate ongoing professional development for pharmacists and foster multidisciplinary collaboration, thus resulting in the provision of high-quality care.

## Conclusions

Implementation strategies and combined implementation strategies–delivery arrangements interventions improved pharmacists' competency in CDM. Most interventions consistently resulted in significant improvements in pharmacists' knowledge, skills, and attitudes. However, one reported no improvement and two reported different results during the follow-up. These findings underscore the potential of tailored, competency-based interventions to enhance pharmacist competencies in chronic disease management while emphasizing the importance of long-term effectiveness. Policymakers can use these findings to create guidelines and policies that promote ongoing professional development for pharmacists.

## Supplementary Information

Supplementary Material 1.

Supplementary Material 2.

Supplementary Material 3.

Supplementary Material 4.

## Data Availability

All the data generated or analyzed during this study are included in this published article [and its supplementary information files].

## Abbreviations

CDM: Chronic Disease Management
EPOC: Effective Practice and Organization of Care
PRISMA: Preferred Reporting Items for Systematic Reviews and Meta-Analyses
RCT: Randomized Controlled Trial
RoB: Risk of Bias
BOPPPS: Bridge-In, Objective, Pre-assessment, Participatory Learning, Postassessment, and Summary
OSCE: Objective Structured Clinical Examination
