# Supplementary material for: Interventions to improve pharmacists’ competency in chronic disease management: a systematic review of randomized controlled trials
Source: BMC Med Educ. 2024 Dec 18;24:1441. doi: 10.1186/s12909-024-06393-z (PMC11654421; doi:10.1186/s12909-024-06393-z)
Supplement: Supplementary file 3 — Supplementary Material 3. [file 12909_2024_6393_MOESM3_ESM.pdf]

Table S3. Risk of bias assessment of randomized controlled trial studies.

**Study**	**Judgment**					
	**D1**	**D2**	**D3**	**D4**	**D5**	**Overall**
Shen et al., 2024	Low	Low	Low	Low	Low	Low
El Hajj et al., 2022	Low	Low	Low	Low	Low	Low
Fujii et al., 2021	Some concerns	Low	Low	Low	Some concerns	Some concerns
Sarayani et al., 2012	Low	Low	Low	Low	Some concerns	Some concerns
Legris et al., 2011	Low	Low	Low	Low	Low	Low
Dolovich et al., 2007	Some concerns	Some concerns	Low	Low	Some concerns	Some concerns
Jackevicius & Chapman, 1999	Low	Some concerns	Low	Low	Low	Some concerns

Domain:
D1 = Bias arising from the randomization process.
D2 = Bias due to deviations from intended intervention.
D3 = Bias due to missing outcome data.
D4 = Bias due to measurement of the outcome.
D5 = Bias in selection of the reported results.
