# Supplementary material for: Interventions to improve pharmacists’ competency in chronic disease management: a systematic review of randomized controlled trials
Source: BMC Med Educ. 2024 Dec 18;24:1441. doi: 10.1186/s12909-024-06393-z (PMC11654421; doi:10.1186/s12909-024-06393-z)
Supplement: Supplementary file 4 — Supplementary Material 4. [file 12909_2024_6393_MOESM4_ESM.pdf]

Table S4. Risk of bias assessment of cluster-randomized controlled trial studies.

Study	Judgment						
	**D1**	**D1b**	**D2**	**D3**	**D4**	**D5**	**Overall**
Haga et al., 2021	Low	Low	Low	Low	Low	Low	Low
Lalonde et al., 2017	Low	Low	Low	Some concerns	Low	Some concerns	Some concerns
Liekens et al., 2014	Some concerns	Low	Low	Low	Low	Some concerns	Some concerns
Lalonde et al., 2008	Low	Low	Low	Some concerns	Some concerns	Some concerns	High

Domain:
D1 = Bias arising from the randomization process.
D1b = Bias arising from the timing of identification and recruitment of individual participants in relation to timing of randomization.
D2 = Bias due to deviations from intended intervention.
D3 = Bias due to missing outcome data.
D4 = Bias due to measurement of the outcome.
D5 = Bias in selection of the reported results.
